# Temporal trends in prevalence and antithrombotic treatment among Asians with atrial fibrillation undergoing percutaneous coronary intervention: A nationwide Korean population-based study

**DOI:** 10.1371/journal.pone.0209593

**Published:** 2019-01-15

**Authors:** Jiesuck Park, Eue-Keun Choi, Kyung-Do Han, You-jung Choi, Euijae Lee, Wonseok Choe, So-Ryoung Lee, Myung-Jin Cha, Woo-Hyun Lim, Jeehoon Kang, Kyung Woo Park, Seil Oh, Gregory Y. H. Lip

**Affiliations:** 1 Department of Internal Medicine, Seoul National University Hospital, Seoul, Republic of Korea; 2 Department of Biostatistics, College of Medicine, The Catholic University of Korea, Seoul, Republic of Korea; 3 Department of Internal Medicine, Soon Chun Hyang University Hospital, Seoul, Republic of Korea; 4 Department of Internal Medicine, Seoul National University Boramae Medical Center, Seoul, Republic of Korea; 5 Institute of Cardiovascular Sciences, University of Birmingham, Birmingham, United Kingdom; 6 Thrombosis Research Unit, Department of Clinical Medicine, Aalborg University, Aalborg, Denmark; Azienda Ospedaliero Universitaria Careggi, ITALY

## Abstract

**Background:**

We investigated the recent 10-year trends in the number of patients with atrial fibrillation (AF) undergoing percutaneous coronary intervention (PCI) in relation to prescription patterns of antithrombotic therapy.

**Methods:**

We analyzed the annual prevalence of PCI and patterns of antithrombotic therapy after PCI, including antiplatelets and oral anticoagulants (vitamin K antagonists and non-vitamin K antagonist oral anticoagulants [NOACs]), in patients with AF between 2006 and 2015 by using the Korean National Health Insurance Service database. Independent factors associated with triple therapy (oral anticoagulant plus dual antiplatelet) prescription were assessed using multivariable logistic regression analysis.

**Results:**

The number of patients with AF undergoing PCI increased gradually from 2006 (n = 2,140) to 2015 (n = 3,631) (p_trend_<0.001). In 2006, only 22.7% of patients received triple therapy after PCI although 96.2% of them were indicated for anticoagulation (CHA_2_DS_2_-VASc score ≥2). The prescription rate of triple therapy increased to 38.3% in 2015 (p_trend_<0.001), which was mainly attributed to a recent increment of NOAC-based triple therapy from 2013 (17.5% in 2015). Previous ischemic stroke or systemic embolism, old age, hypertension, and congestive heart failure were significantly associated with a higher triple therapy prescription rate, whereas previous myocardial infarction, PCI, and peripheral arterial disease were associated with triple therapy underuse.

**Conclusions:**

From 2006 to 2015, the number of patients with AF undergoing PCI and the prescription rate of triple therapy increased gradually with a recent increment of NOAC-based antithrombotic therapy from 2013. Previous myocardial infarction, peripheral artery disease, and PCI were associated with underuse of triple therapy.

## Introduction

Atrial fibrillation (AF) is a disorder with an increasing prevalence. Because it confers significant cardiovascular morbidity and mortality from stroke and thromboembolism [1], the guidelines recommend oral anticoagulants (OACs) as a preventive strategy in patients with AF with a moderate to high risk of developing stroke [2–4]. Among patients with AF, 5–15% are known to undergo percutaneous coronary intervention (PCI) during their entire lifespan [2], and approximately 5–10% of patients undergoing PCI have concomitant AF [5]. Patients with coronary artery disease (CAD) undergoing PCI and stent implantation need to be treated with dual antiplatelet agents (aspirin and P2Y_12_ antagonists) to reduce the risk of stent thrombosis. Therefore, patients with AF undergoing PCI and stent implantation are recommended to receive combination antithrombotic therapy including OACs and antiplatelets. However, there are concerns about the clinical benefit of intensive combination antithrombotic therapy, as it increases the risk of fatal bleeding, which would offset the benefit in reducing the ischemic risk [6–9]. As non-vitamin K antagonist oral anticoagulants (NOACs) had shown a safety benefit in patients with AF compared with conventional vitamin K antagonists (VKAs) [10,11], recent clinical trials have investigated the clinical benefit of dual antithrombotic therapy combining NOAC and P2Y_12_ antagonists (mostly clopidogrel) as an alternative to triple therapy after PCI in patients with AF [12–15]. However, there is a paucity of data providing real-world evidence for the prescription patterns of antithrombotic therapy among patients with AF after PCI, especially among the Asian population. Therefore, we investigated the recent 10-year trends in the number of patients with AF undergoing PCI in relation to antithrombotic therapy by using the Korean National Health Insurance database.

## Materials and methods

### Study population and data source

The study population and clinical data were extracted from the National Health Claims Database established by the Korean National Health Insurance Service (NHIS) [16,17]. The NHIS system provides comprehensive medical care coverage to 97% of the Korean population, and maintains a database comprising inpatient and outpatient medical service records, diagnostic and procedural codes, prescribed medication claims, and patients’ demographic data. The Medical Aid program covers the low-income population (the remaining 3%). From 2006, the clinical data recorded by the Medical Aid program have been included in the NHIS database; thus, the NHIS claims database represents the entire Korean population. On the basis of the International Classification of Disease, Tenth Revision, Clinical Modification (ICD-10-CM) codes, we analyzed the data of all Koreans aged ≥20 years between January 2006 and December 2015. This study was exempt from review by the Seoul National University Hospital Institutional Review Board (1706-160-863) and was conducted according to the tenets of the Declaration of Helsinki.

### Definitions of non-valvular atrial fibrillation, comorbidities, and percutaneous coronary intervention

Non-valvular AF was defined as in previous reports [1,18,19]. Patients diagnosed as having AF during hospitalization or those with ≥2 diagnoses in outpatient clinics were included. The diagnostic codes for AF were I480–I484 and I489 (ICD-10-CM codes). Patients with mitral stenosis (I50, I52, and I59) or mechanical heart valves (Z952–Z954) were excluded. The detailed definitions of comorbidities are summarized in S1 Table. Briefly, hypertension and diabetes mellitus were defined based on a combination of diagnostic codes and the use of at least 1 antihypertensive or antidiabetic drug, respectively. Heart failure, previous stroke or thromboembolism, myocardial infarction (MI), peripheral artery disease (PAD), and intracranial hemorrhage (ICH) were defined based on ICD-10-CM codes [1,18,19]. To assess the stroke risk of individual patients, we calculated the CHA_2_DS_2_-VASc scores by assigning 1 point for age between 65 and 74 years, female sex, and the presence of hypertension, diabetes mellitus, heart failure, and vascular disease (previous MI or PAD), and 2 points for previous stroke/transient ischemic attack/thromboembolism or age ≥75 years [20]. Patients with a high stroke risk were defined as those with a CHA_2_DS_2_-VASc score of ≥2. The procedure codes for PCI (M6561, M6562, M6563, and M6564) were used for patients who had undergone PCI during the study period. For those who had undergone multiple PCI procedures in separate years, each procedure was counted separately.

### Antithrombotic therapy

We reviewed the inpatient and outpatient prescription records of antithrombotic therapy including aspirin, clopidogrel, VKAs, and NOACs annually from 2006 to 2015. However, as edoxaban was introduced in Korea only in 2016, it was not included under the NOACs regimen in our analysis. Additionally, newer P2Y_12_ inhibitors such as prasugrel and ticagrelor were not included in our analysis because they were not widely available in Korea during our study period. According to the prescription of antithrombotic therapy, patients were categorized into the following groups: no treatment, single antiplatelet therapy (SAPT), OAC monotherapy, dual antiplatelet therapy (DAPT), dual therapy with OAC and SAPT, VKA-based triple therapy, and NOAC-based triple therapy.

### Statistical analysis

The total numbers of patients with AF and those who underwent PCI, the total number of PCI procedures, and the prescription regimen of antithrombotic therapy after PCI were investigated on a yearly basis. The baseline characteristics of the study population including demographic information and comorbidities were also investigated annually. Categorical variables were presented as numbers and percentages, whereas continuous data were described as means with standard deviations. The significance of linear time trends over the entire study period was assessed using logistic and linear regression models. Subgroup analyses for the trends in prescription patterns of antithrombotic therapy were performed according to sex and for patients at a high risk of stroke (CHA_2_DS_2_-VASc score ≥2).

To investigate the clinical factors associated with underuse of triple therapy after PCI in patients with AF, baseline characteristics were compared between patients with triple therapy and those with only DAPT after PCI among the total population in 2015. Multivariate logistic regression analysis was applied to identify factors independently associated with underuse of triple therapy. Trends in the prescription rate of triple therapy according to CHA_2_DS_2_-VASc scores were assessed using logistic regression analysis. Statistical significance was set at two-tailed p-values of <0.05. Statistical analyses were performed using SAS software version 9.3 (SAS Institute, Cary, NC, USA).

## Results and discussion

### Baseline characteristics of the study population

**Fig 1:**
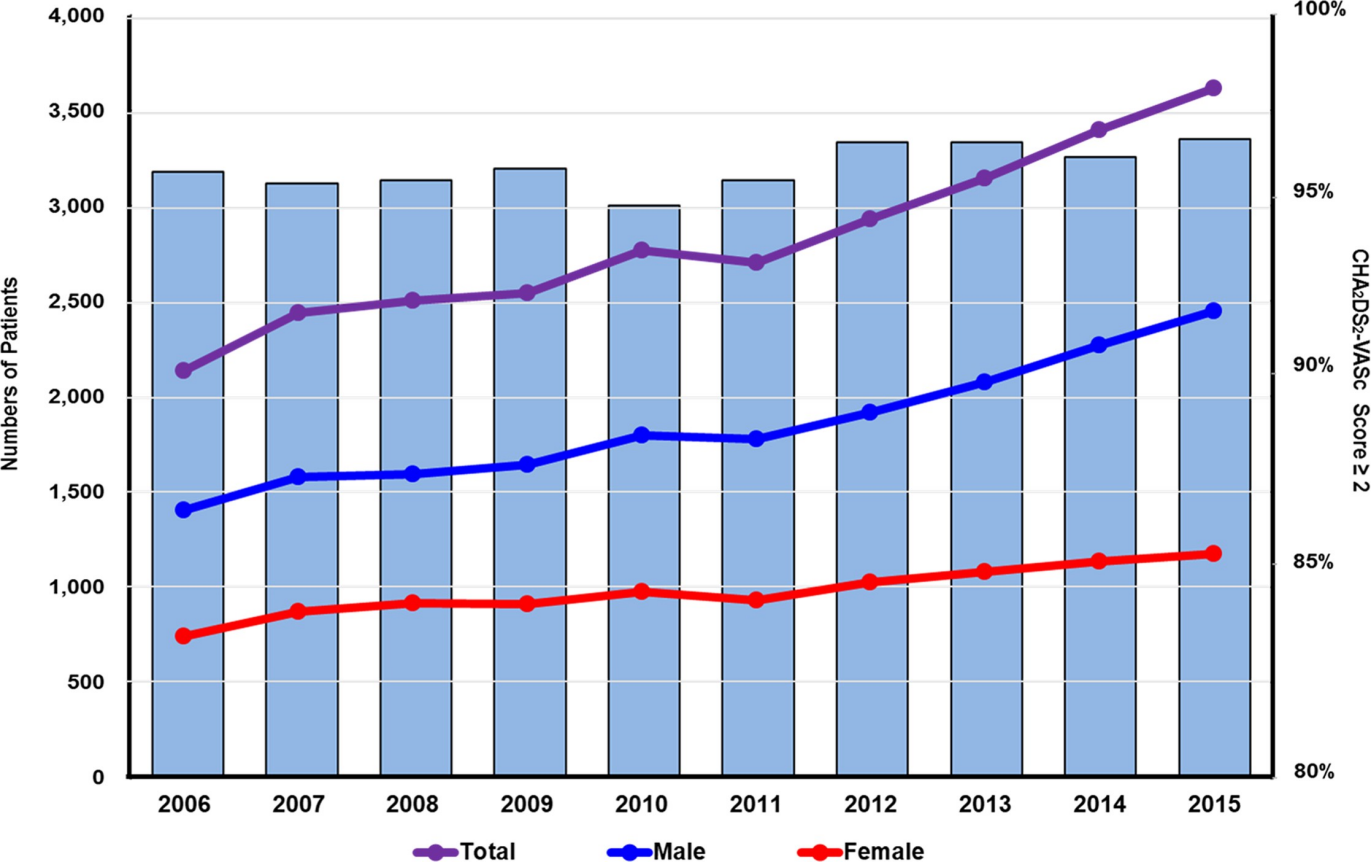
The 10-year trends in the number of patients with atrial fibrillation undergoing percutaneous coronary intervention. The number of patients with AF undergoing PCI steadily increased from 2006 to 2015 (p_trend_<0.001) regardless of sex. In each year, >90% of patients were at a high risk of stroke (CHA2DS2-VASc score ≥2). AF, atrial fibrillation; PCI, percutaneous coronary intervention.

The total number of patients with AF undergoing PCI increased gradually from 2,140 in 2006 to 3,631 in 2015 (p_trend_<0.001), although the number of PCI procedures per patient decreased slightly (1.19±0.42 in 2006 to 1.16±0.40 in 2015) (Table 1 and Fig 1). Among patients who had undergone PCI in 2015 (n = 53,280), approximately 7% had concomitant AF. The mean patient age increased from 66.3±10.5 years in 2006 to 70.8±10.5 years in 2015 (p_trend_<0.001), with the percentage of those aged ≥75 years being 41.3% in 2015. Approximately 60% of patients throughout the study period were men. The prevalence of diabetes mellitus and congestive heart failure increased during 10 years (40.3% in 2015 for diabetes mellitus [p_trend_<0.001] and 58.3% in 2015 for congestive heart failure [p_trend_<0.001]) (Table 1 and S1 Fig). In each year, >90% of the patients were at a high risk of stroke (CHA_2_DS_2_-VASc score ≥2).

**Table 1:** Baseline characteristics of the study population.

Years	2006	2007	2008	2009	2010	2011	2012	2013	2014	2015	p_trend_
**Total AF patients**	124549	137604	150301	163943	177663	185205	205813	225834	248925	276842	<0.001
**AF patients with PCI**	2140 (1.7)	2446 (1.8)	2511 (1.7)	2553 (1.6)	2778 (1.6)	2713 (1.5)	2940 (1.4)	3157 (1.4)	3411 (1.4)	3631 (1.3)	<0.001
**Total PCI procedure**	2541	2933	2985	3026	3248	3200	3443	3694	3932	4220	<0.001
**Number of PCI per patient**	1.19±0.42	1.20±0.43	1.19±0.41	1.19±0.41	1.17±0.39	1.18±0.40	1.17±0.39	1.17±0.39	1.15±0.37	1.16±0.40	<0.001
**Demographics**											
**Age, years**	66.3±10.5	67.1±10.3	67.6±10.4	68.9±10.0	69.0±10.2	70.1±9.8	70.2±10.3	70.1±10.4	70.5±10.8	70.8±10.5	<0.001
**Age**											
65–74 years	839 (39.2)	974 (39.8)	1023 (40.7)	1047 (41.0)	1097 (39.5)	1050 (38.7)	1079 (36.7)	1122 (35.5)	1124 (33.0)	1187 (32.7)	
75 years ≤	472 (22.1)	589 (24.1)	672 (26.8)	790 (30.9)	878 (31.6)	988 (36.4)	1120 (38.1)	1174 (37.2)	1376 (40.3)	1501 (41.3)	
**Male**	1403 (65.6)	1578 (64.5)	1595 (63.5)	1646 (64.5)	1802 (64.9)	1782 (65.7)	1918 (65.2)	2078 (65.8)	2277 (66.8)	2457 (67.7)	<0.001
**Comorbidities**											
Diabetes mellitus	740 (34.6)	850 (34.8)	881 (35.1)	944 (37.0)	1015 (36.5)	1038 (38.3)	1136 (38.6)	1242 (39.3)	1289 (37.8)	1463 (40.3)	<0.001
Hypertension	1984 (92.7)	2239 (91.5)	2296 (91.4)	2297 (90.0)	2457 (88.4)	2364 (87.1)	2566 (87.3)	2779 (88.0)	2951 (86.5)	3098 (85.3)	0.012
Dyslipidemia	1776 (83.0)	2030 (83.0)	2040 (81.2)	2060 (80.7)	2215 (79.7)	2151 (79.3)	2429 (82.6)	2606 (82.6)	2958 (86.7)	3121 (86.0)	<0.001
Congestive heart failure	679 (31.7)	690 (28.2)	791 (31.5)	876 (34.3)	1094 (39.4)	1259 (46.4)	1401 (47.7)	1617 (51.2)	1900 (55.7)	2118 (58.3)	<0.001
Myocardial infarction	1207 (56.4)	1287 (52.6)	1209 (48.2)	1201 (47.0)	1242 (44.7)	1079 (39.8)	1194 (40.6)	1330 (42.1)	1491 (43.7)	1625 (44.8)	<0.001
Peripheral arterial disease	509 (23.8)	518 (21.2)	637 (25.3)	694 (27.2)	664 (23.9)	641 (23.6)	691 (23.5)	726 (23.0)	784 (23.0)	884 (24.4)	<0.001
Stroke/TIA/thromboembolism	613 (28.6)	736 (30.1)	685 (27.3)	710 (27.8)	799 (28.8)	820 (30.2)	912 (31.0)	927 (29.4)	993 (29.1)	1071 (29.5)	<0.001
Intracranial hemorrhage	53 (2.5)	48 (2.0)	57 (2.3)	66 (2.6)	86 (3.1)	80 (3.0)	89 (3.0)	134 (4.2)	132 (3.9)	97 (2.7)	<0.001
**CHA**_**2**_**DS**_**2**_**-VASc Score**	4.3±1.8	4.3±1.8	4.3±1.8	4.5±1.8	4.4±1.9	4.6±1.9	4.7±1.9	4.7±1.9	4.8±1.9	4.8±1.9	<0.001
Score 2 ≤	2058 (96.2)	2345 (95.9)	2410 (96.0)	2458 (96.3)	2646 (95.3)	2604 (96.0)	2851 (97.0)	3063 (97.0)	3294 (96.6)	3528 (97.1)	<0.001

Abbreviations: AF, atrial fibrillation; PCI, percutaneous coronary intervention; TIA, transient ischemic attack

Values are given as mean±standard deviation or number (percentage), unless otherwise indicated.

### Ten-year temporal trends in antithrombotic treatment regimens in patients with atrial fibrillation undergoing percutaneous coronary intervention

**Fig 2:**
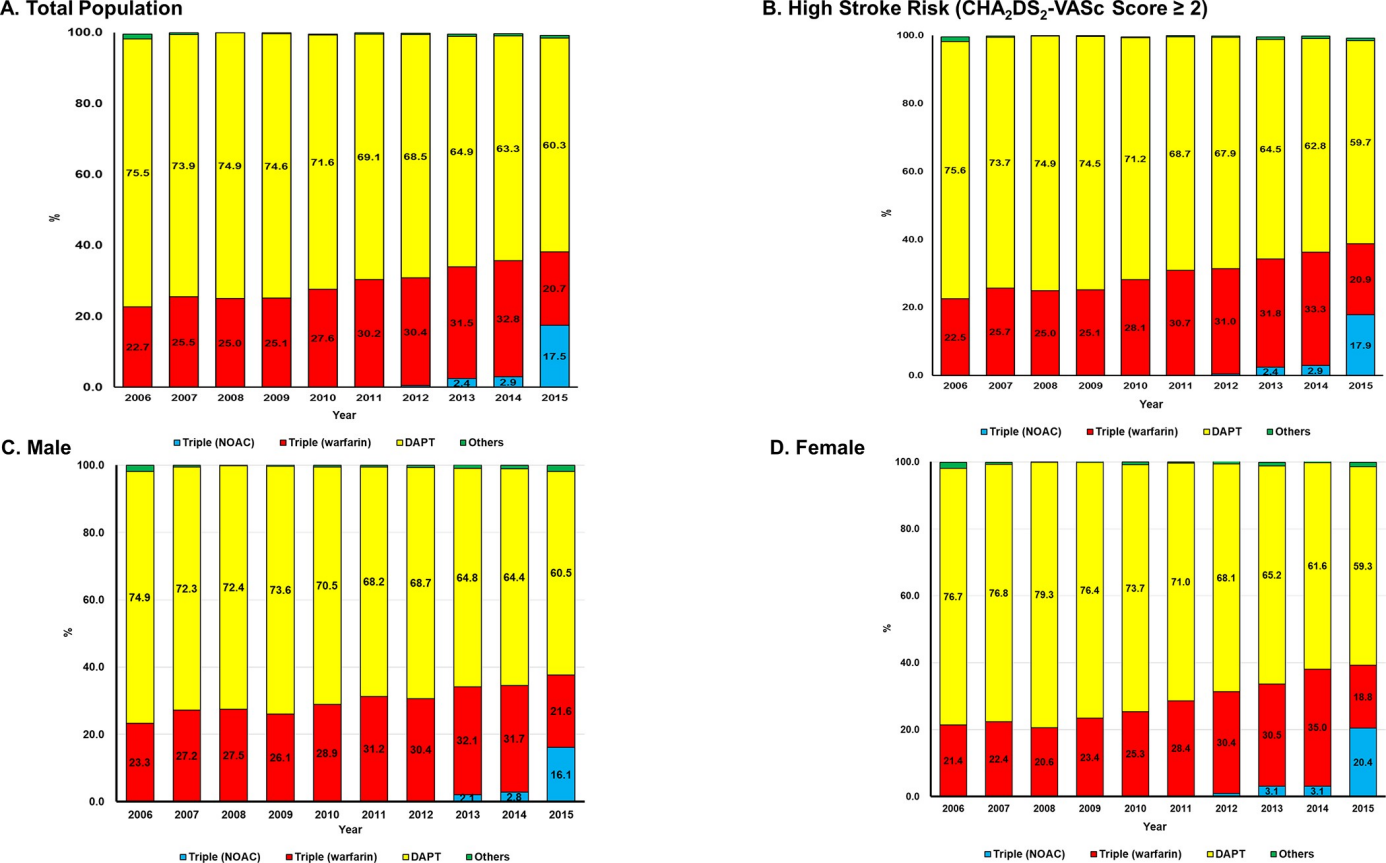
Temporal trends in antithrombotic treatment in patients with atrial fibrillation undergoing percutaneous coronary intervention. The 10-year trends in prescription patterns of antithrombotic therapy among patients with AF undergoing PCI are presented among the total population (a), those at high risk of stroke (CHA2DS2-VASc score ≥2) (b), and both male (c) and female (d) patients. Patients who received SAPT, OAC monotherapy, and dual therapy with OAC and SAPT were grouped into other types of antithrombotic therapy, as the prescription rates were very low. AF, atrial fibrillation; PCI, percutaneous coronary intervention; DAPT, dual antiplatelet therapy; NOAC, non-vitamin K antagonist oral anticoagulants.

In 2006, 75.5% of patients received DAPT, whereas only 22.7% were prescribed triple therapy with VKAs. However, the percentage of those with triple therapy increased to 38.3% in 2015 (p_trend_<0.001), which was mainly attributed to a recent increment of NOAC-based triple therapy from 2013, comprising 45.8% of the overall triple therapy in 2015 (p_trend_<0.001) (Table 2 and Fig 2A). These trends were also similar among patients at a high risk of stroke (38.8% for triple therapy in 2015) (Fig 2B), and in both sexes (37.7% and 39.2% for triple therapy in 2015 for male and female patients, respectively) (Fig 2C and 2D).

**Table 2:** Temporal trends of prescription of antithrombotic therapy among patients with atrial fibrillation undergoing percutaneous coronary intervention.

**Overall population**											
**Year**	**2006**	**2007**	**2008**	**2009**	**2010**	**2011**	**2012**	**2013**	**2014**	**2015**	**p**_**trend**_
**Total patients**	**2140**	**2446**	**2511**	**2553**	**2778**	**2713**	**2940**	**3157**	**3411**	**3631**	<0.001
SAPT	29 (1.4)	10 (0.4)	2 (0.1)	6 (0.2)	6 (0.2)	11 (0.4)	11 (0.4)	22 (0.7)	25 (0.7)	25 (0.7)	0.374
DAPT	1616 (75.5)	1808 (73.9)	1880 (74.9)	1904 (74.6)	1990 (71.6)	1876 (69.1)	2014 (68.5)	2050 (64.9)	2159 (63.3)	2188 (60.3)	<0.001
OAC monotherapy	0 (0.0)	0 (0.0)	0 (0.0)	0 (0.0)	1 (0.0)	0 (0.0)	1 (0.0)	0 (0.0)	0 (0.0)	0 (0.0)	0.974
Dual therapy (OAC+APT)	10 (0.5)	4 (0.2)	2 (0.1)	2 (0.1)	13 (0.5)	2 (0.1)	7 (0.2)	14 (0.4)	10 (0.3)	31 (0.9)	0.006
Triple (warfarin)	485 (22.7)	624 (25.5)	627 (25.0)	641 (25.1)	767 (27.6)	820 (30.2)	895 (30.4)	995 (31.5)	1118 (32.8)	752 (20.7)	<0.001
Triple (NOAC)	0 (0.0)	0 (0.0)	0 (0.0)	0 (0.0)	1 (0.0)	3 (0.1)	12 (0.4)	76 (2.4)	99 (2.9)	635 (17.5)	<0.001
**High stroke risk**^a^											
**Year**	**2006**	**2007**	**2008**	**2009**	**2010**	**2011**	**2012**	**2013**	**2014**	**2015**	**p**_**trend**_
**Total patients**	**2058**	**2345**	**2410**	**2458**	**2646**	**2604**	**2851**	**3063**	**3294**	**3528**	<0.001
SAPT	28 (1.4)	9 (0.4)	2 (0.1)	6 (0.2)	6 (0.2)	10 (0.4)	10 (0.4)	22 (0.7)	24 (0.7)	23 (0.7)	0.449
DAPT	1556 (75.6)	1729 (73.7)	1804 (74.9)	1832 (74.5)	1884 (71.2)	1788 (68.7)	1937 (67.9)	1977 (64.5)	2068 (62.8)	2106 (59.7)	<0.001
OAC monotherapy	0 (0.0)	0 (0.0)	0 (0.0)	0 (0.0)	1 (0.0)	0 (0.0)	1 (0.0)	0 (0.0)	0 (0.0)	0 (0.0)	0.979
Dual therapy (OAC+APT)	10 (0.5)	4 (0.2)	2 (0.1)	2 (0.1)	11 (0.4)	2 (0.1)	7 (0.2)	14 (0.5)	9 (0.3)	31 (0.9)	0.006
Triple (warfarin)	464 (22.5)	603 (25.7)	602 (25.0)	618 (25.1)	743 (28.1)	800 (30.7)	884 (31.0)	975 (31.8)	1096 (33.3)	737 (20.9)	<0.001
Triple (NOAC)	0 (0.0)	0 (0.0)	0 (0.0)	0 (0.0)	1 (0.0)	3 (0.1)	12 (0.4)	75 (2.4)	97 (2.9)	631 (17.9)	<0.001
**Male**											
**Year**	**2006**	**2007**	**2008**	**2009**	**2010**	**2011**	**2012**	**2013**	**2014**	**2015**	**p**_**trend**_
**Total patients**	**1403**	**1578**	**1595**	**1646**	**1802**	**1782**	**1918**	**2078**	**2277**	**2457**	<0.001
SAPT	18 (1.3)	6 (0.4)	2 (0.1)	5 (0.3)	4 (0.2)	7 (0.4)	9 (0.5)	13 (0.6)	19 (0.8)	19 (0.8)	0.206
DAPT	1051 (74.9)	1141 (72.3)	1154 (72.4)	1211 (73.6)	1271 (70.5)	1215 (68.2)	1318 (68.7)	1346 (64.8)	1466 (64.4)	1486 (60.5)	<0.001
OAC monotherapy	0 (0.0)	0 (0.0)	0 (0.0)	0 (0.0)	0 (0.0)	0 (0.0)	0 (0.0)	0 (0.0)	0 (0.0)	0 (0.0)	-
Dual therapy (OAC+APT)	7 (0.5)	1 (0.1)	1 (0.1)	1 (0.1)	7 (0.4)	2 (0.1)	4 (0.2)	10 (0.5)	7 (0.3)	25 (1.0)	0.002
Triple (warfarin)	327 (23.3)	430 (27.2)	438 (27.5)	429 (26.1)	520 (28.9)	556 (31.2)	584 (30.4)	666 (32.1)	721 (31.7)	531 (21.6)	0.001
Triple (NOAC)	0 (0.0)	0 (0.0)	0 (0.0)	0 (0.0)	0 (0.0)	1 (0.1)	3 (0.2)	43 (2.1)	64 (2.8)	396 (16.1)	<0.001
**Female**											
**Year**	**2006**	**2007**	**2008**	**2009**	**2010**	**2011**	**2012**	**2013**	**2014**	**2015**	**p**_**trend**_
**Total patients**	**737**	**868**	**916**	**907**	**976**	**931**	**1022**	**1079**	**1134**	**1174**	<0.001
SAPT	11 (1.5)	4 (0.5)	0 (0.0)	1 (0.1)	2 (0.2)	4 (0.4)	2 (0.2)	9 (0.8)	6 (0.5)	6 (0.5)	0.735
DAPT	565 (76.7)	667 (76.8)	726 (79.3)	693 (76.4)	719 (73.7)	661 (71.0)	696 (68.1)	704 (65.2)	693 (61.6)	702 (59.8)	<0.001
OAC monotherapy	0 (0.0)	0 (0.0)	0 (0.0)	0 (0.0)	1 (0.1)	0 (0.0)	1 (0.1)	0 (0.0)	0 (0.0)	0 (0.0)	0.743
Dual therapy (OAC+APT)	3 (0.4)	3 (0.3)	1 (0.1)	1 (0.1)	6 (0.6)	0 (0.0)	3 (0.3)	4 (0.4)	3 (0.3)	6 (0.5)	0.685
Triple (warfarin)	158 (21.4)	194 (22.4)	189 (20.6)	212 (23.4)	247 (25.3)	264 (28.4)	311 (30.4)	329 (30.5)	397 (35.0)	221 (18.8)	<0.001
Triple (NOAC)	0 (0.0)	0 (0.0)	0 (0.0)	0 (0.0)	1 (0.1)	2 (0.2)	9 (0.9)	33 (3.1)	35 (3.1)	239 (20.4)	<0.001

Abbreviations: APT, antiplatelet; DAPT, dual antiplatelet therapy; NOAC, non-vitamin K antagonist oral anticoagulant; OAC, oral anticoagulant; SAPT single antiplatelet therapy

Values are given as number (percentage), unless otherwise indicated.

^a^CHA2DS2-VASc score ≥2

### Risk factors associated with underuse of triple antithrombotic therapy

**Fig 3:**
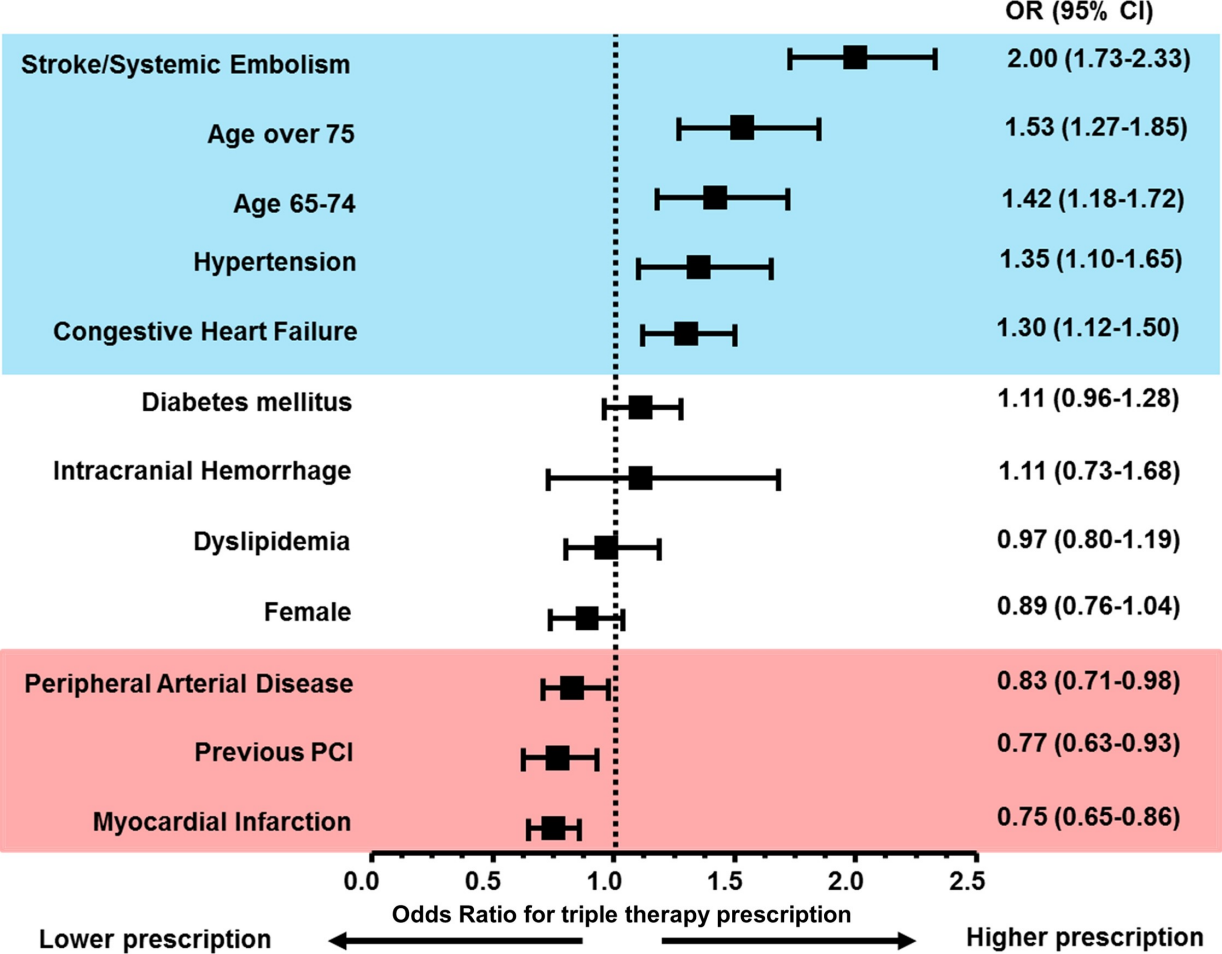
Factors associated with prescriptions of triple therapy. History of stroke or systemic embolism, old age (>65 years), hypertension, and congestive heart failure were significantly associated with higher prescriptions of triple therapy after PCI. However, histories of peripheral artery disease, PCI, and myocardial infarction were associated with underuse of triple therapy. CI, confidence interval; OR, odds ratio; PCI, percutaneous coronary intervention.

With respect to factors associated with prescription of triple therapy after PCI, a history of stroke or systemic embolism, old age (>65 years), hypertension, and congestive heart failure were independently associated with a higher prescription rate of triple therapy (S2 Table and Fig 3). However, a history of MI, PCI, and PAD was associated with underuse of triple therapy after PCI.

### Trends in patients with different risks of stroke

An incremental relationship was observed between CHA_2_DS_2_-VASc scores and prescription of triple therapy (S2 Fig). Patients with a CHA_2_DS_2_-VASc score of ≥4 showed significantly higher odds for receiving triple therapy after PCI than those with a score of 0 as a reference (odds ratio [OR] 3.62, 95% confidence interval [CI] 1.06–12.41 for patients with CHA_2_DS_2_-VASc scores of 4).

In this large population-based cohort study, our principal findings were as follows: (1) the number of patients with AF undergoing PCI increased in the recent 10 years; (2) most patients with AF undergoing PCI were administered DAPT without concomitant OAC prescription; (3) triple therapy prescription showed a gradual increase over time, being used in approximately one-third of patients with AF undergoing PCI, particularly after the approval of NOAC in 2013; (4) previous MI, PCI, and PAD were associated with underuse of triple therapy; and (5) there was an incremental relationship between CHA_2_DS_2_-VASc scores and triple therapy prescriptions. To our knowledge, this is the first and largest Asian study to investigate the temporal trends in the number of patients with AF undergoing PCI in relation to antithrombotic therapy.

### Prevalence of atrial fibrillation in patients undergoing percutaneous coronary intervention

Considering the increasing incidence and prevalence of AF [1], the number of patients with AF undergoing PCI will also increase. Indeed, we observed that approximately 7% of patients who underwent PCI had AF in 2015, consistent with previous studies [5,21–24]. For example, a recent Asian study reported that 7% of patients presented with a diagnosis of AF at the index PCI, and this was associated with an increased risk of ischemia and bleeding complications [5]. Additionally, patients with AF generally show more severe comorbidities and more complex CAD at baseline, leading to higher mortality and a higher rate of cardiovascular events [25,26]. In our results, the mean CHA_2_DS_2_-VASc score of the patients increased to 4.8±1.9 in 2015, and >90% of patients each year had a CHA_2_DS_2_-VASc score of ≥2, which were higher than those reported in previous studies [5,7]. Although the complexity of CAD was not evaluated in our study, these results would reflect the higher burden of cardiovascular risk factors and the severity of disease in our study population.

### Antithrombotic therapy in patients with atrial fibrillation undergoing percutaneous coronary intervention in real-world clinical practice

The current guidelines recommend combination antithrombotic therapy in patients with AF after PCI with stent implantation [2,3,26,27]. Although the optimal duration and treatment regimens remain under debate, OAC is considered an essential component in any combination. However, concomitant prescription of antiplatelets with OAC predisposes patients to a higher risk of bleeding [7], which could result in early discontinuation of antithrombotic therapy, leading to a higher risk of stent thrombosis. Although non-Asians were reported to have a lower risk of bleeding associated with the use of OAC than Asians [28], underprescription of OAC after PCI has also been observed in non-Asian populations. Among the 1648 patients with AF with non-ST segment elevation MI who underwent PCI in the CRUSADE (Can Rapid risk stratification of Unstable angina patients Suppress ADverse outcomes with Early implementation of the American College of Cardiology/American Heart Association guidelines) registry, only 27% (n = 448) of the total population received triple therapy at discharge [29]. Among 12,165 Danish patients with AF concomitant with acute MI or PCI between 2001 and 2009, only 15% (n = 1,895) received triple therapy [30]. This number is even lower in the Asian population, with a recent registry reporting that only 10% of patients with AF who had PCI and drug-eluting stent implantation received triple therapy at discharge [5]. In the current nationwide study, we found that the prescription rate of triple therapy showed a gradual increment in the recent 10 years. Nevertheless, the percentage of patients receiving triple therapy after PCI was only 38.2% in 2015. Previously, we reported that female sex, prior vascular disease, and ICH were associated with underuse of OAC in the entire Korean population with AF [31]. Similarly, in the current study, a history of vascular disease such as MI or PAD, and a previous PCI event were associated with underuse of triple therapy in patients with AF after PCI. We inferred that the requirement for antiplatelet therapy in these patients would discourage physicians from combining OAC with antiplatelets after PCI. These trends could be related to concerns about the increased bleeding risk when prescribing OAC among patients with MI or those receiving antiplatelet therapy [32]. However, Pastori et al. [33] have shown a significantly higher risk of adverse cardiovascular events in patients with AF with concomitant PAD (hazard ratio [HR] 1.8, 95% CI 1.1–2.9), CAD (HR 2.2, 95% CI 1.4–3.4), or both (HR 2.4, 95% CI 1.4–4.4) than in those without any risk factors, suggesting that more optimized antithrombotic therapy is necessary for these patients. Interestingly, we found a significant increase in the prescription of triple therapy after PCI among patients with AF who had a CHA_2_DS_2_-VASc score of ≥4. This result also implies that the threshold for the initiation of OAC in clinical practice is much higher for patients with AF after PCI than in the general AF population.

### Role of non-vitamin K antagonist oral anticoagulants in antithrombotic therapy among patients with atrial fibrillation undergoing percutaneous coronary intervention

As NOACs had shown clinical benefits compared with VKAs among patients with AF, clinical trials investigating the benefit of NOAC-based antithrombotic therapy in patients with AF undergoing PCI have been conducted [12,13]. The PIONEER AF-PCI (Open-label, Randomized, Controlled, Multicenter Study Exploring Two Treatment Strategies of Rivaroxaban and a Dose-Adjusted Oral Vitamin K Antagonist Treatment Strategy in Subjects with Atrial Fibrillation Who Undergo Percutaneous Coronary Intervention) trial [12] showed that the rates of clinically significant bleeding episodes were lower in patients administered NOAC-based antithrombotic therapy (rivaroxaban 15 mg once daily combined with a P2Y_12_ inhibitor over 12 months or 2.5 mg twice daily combined with DAPT) than in those administered VKA-based triple therapy (16.8%, 18.0%, and 26.7%, respectively). Additionally, the RE-DUAL (Randomized Evaluation of Dual Antithrombotic Therapy with Dabigatran vs. Triple Therapy with Warfarin in Patients with Non-valvular Atrial Fibrillation Undergoing Percutaneous Coronary Intervention) trial [13] compared the safety outcomes between dabigatran-based antithrombotic therapy and VKA-based triple therapy in patients with AF undergoing PCI, and showed that those receiving dabigatran had significantly lower rates of subsequent bleeding risks. Furthermore, 2 randomized clinical trials investigating the safety and efficacy of antithrombotic therapy based on apixaban [14] or edoxaban [15] combined with P2Y_12_ inhibitors vs. triple therapy with conventional VKA are ongoing. Of note, the recent European Society of Cardiology guidelines have been updated and recommend the use of dual therapy with OAC plus clopidogrel 75 mg/day as an alternative to 1-month triple antithrombotic therapy in patients in whom the risk of bleeding outweighs the risk of ischemia [4].

### Limitations

While this study covers nationwide data of the entire Korean population, some limitations are evident. Information about individual patients’ drug compliance, clinical information about contraindications to OACs use based on the assessment of bleeding risk, and laboratory data including time in therapeutic range in patients using VKAs were not available. The current study included prescription records of antithrombotic therapy until 2015; therefore, the trends from 2016 cannot be presented. However, as the current study showed an incremental trend of NOAC-based antithrombotic therapy especially from 2013, we can presume an even higher prescription rate after 2016. Finally, the long-term clinical efficacy and safety outcomes in patients with AF undergoing PCI according to the different antithrombotic therapy regimens were not included in our results and need to be further investigated.

## Conclusions

From 2006 to 2015, an increasing trend in the number of patients with AF undergoing PCI was observed. The prescription rate of triple therapy after PCI had increased gradually, despite remaining <40%, with a recent increment of NOAC-based antithrombotic therapy from 2013. Previous MI, PAD, and PCI were associated with underuse of triple therapy.

## Supporting information

S1 Table Definitions of comorbidities. (PDF)

S2 Table Factors associated with underuse of triple therapy among patients with atrial fibrillation undergoing percutaneous coronary intervention. (PDF)

S1 Fig Temporal trends in baseline characteristics of the study population. (PDF)

S2 Fig Trends in triple therapy prescriptions based on the CHA2DS2-VASc score. CI, confidence interval; OR, odds ratio
(PDF)
