# Supplementary material for: Temporal trends in prevalence and antithrombotic treatment among Asians with atrial fibrillation undergoing percutaneous coronary intervention: A nationwide Korean population-based study
Source: PLoS One. 2019 Jan 15;14(1):e0209593. doi: 10.1371/journal.pone.0209593 (PMC6333333; doi:10.1371/journal.pone.0209593)
Supplement: S1 Table — (PDF) [file pone.0209593.s001.pdf]

S1 Table.

Diagnosis	ICD-10-CM code and definition
Hypertension[a]	I10-I13, I15; and minimum 1 prescription of anti-hypertensive drug (thiazide, loop diuretics, aldosterone antagonist, alpha-/beta-blocker, calcium-channel blocker, angiotensin-converting enzyme inhibitor, angiotensin II receptor blocker).
Diabetes mellitus[a]	E11-E14; and minimum 1 prescription of anti-diabetic drugs (sulfonylureas, metformin, meglitinides, thiazolidinediones, dipeptidyl peptidase-4 inhibitors, α-glucosidase inhibitors and insulin).
Dyslipidemia	E78
Congestive Heart failure	I50
Stroke	I63, I64
Transient ischemic attack	G458, G459
Systemic thromboembolism	I26, I74, I802
Intracranial hemorrhage	I60-I62
Vascular disease	
Previous myocardial infarction	I21, I22
Peripheral artery disease	I70, I73

All variables except hypertension and diabetes mellitus are defined when patients had one or more diagnoses during hospitalization or at outpatient clinic.

[a] Hypertension and diabetes mellitus were identified when patients had ≥1 diagnoses during hospitalization or ≥2 diagnoses at outpatient clinic for preventing overestimation of diagnosis.
