# Supplementary material for: Temporal trends in prevalence and antithrombotic treatment among Asians with atrial fibrillation undergoing percutaneous coronary intervention: A nationwide Korean population-based study
Source: PLoS One. 2019 Jan 15;14(1):e0209593. doi: 10.1371/journal.pone.0209593 (PMC6333333; doi:10.1371/journal.pone.0209593)
Supplement: S2 Table — (PDF) [file pone.0209593.s002.pdf]

S2 Table.

	DAPT Only (n=2188)	Triple Therapy (n=1387)	Multivariate OR (95% CI)
**Age**			
≤ 64	646 (29.5)	287 (20.7)	1
65 - 74	691 (31.6)	472 (34.0)	1.42 (1.18-1.72)
75 ≤	851 (38.9)	628 (45.3)	1.53 (1.27-1.85)
**Sex**			
Male	1486 (67.9)	927 (66.8)	1
Female	702 (32.1)	460 (33.2)	0.89 (0.76-1.04)
**Diabetes Mellitus**			
No	1336 (61.1)	804 (58.0)	1
Yes	852 (38.9)	583 (42.0)	1.11 (0.96-1.28)
**Hypertension**			
No	360 (16.5)	167 (12.0)	1
Yes	1828 (83.6)	1220 (88.0)	1.35 (1.10-1.65)
**Dyslipidemia**			
No	301 (13.8)	200 (14.4)	1
Yes	1887 (86.2)	1187 (85.6)	0.97 (0.80-1.19)

**Congestive Heart Failure**			
No	968 (44.2)	518 (37.3)	1
Yes	1220 (55.8)	869 (62.7)	1.30 (1.12-1.50)
**Myocardial Infarction**			
No	1148 (52.5)	813 (58.6)	1
Yes	1040 (47.5)	574 (41.4)	0.75 (0.65-0.86)
**Peripheral Arterial Disease**			
No	1650 (75.4)	1056 (76.1)	1
Yes	538 (24.6)	331 (23.9)	0.83 (0.71-0.98)
**Stroke/TIA/Thromboembolism**			
No	1676 (76.6)	843 (60.8)	1
Yes	512 (23.4)	544 (39.2)	2.00 (1.73-2.33)
**Intracranial Hemorrhage**			
No	2134 (97.5)	1344 (96.9)	1
Yes	54 (2.5)	43 (3.1)	1.11 (0.73-1.68)
**Previous History of PCI**			
No	1809 (82.7)	1194 (86.1)	1
Yes	379 (17.3)	193 (13.9)	0.77 (0.63-0.93)

Abbreviation: CI, confidence interval; DAPT, dual antiplatelet agents; OR, odds ratio; PCI, percutaneous coronary intervention; TIA, transient ischemic attack.

Values given as number (percentage), unless otherwise indicated.
