# Supplementary figures and images for: Temporal trends in prevalence and antithrombotic treatment among Asians with atrial fibrillation undergoing percutaneous coronary intervention: A nationwide Korean population-based study
Source: PLoS One. 2019 Jan 15;14(1):e0209593. doi: 10.1371/journal.pone.0209593 (PMC6333333; doi:10.1371/journal.pone.0209593)

S1 Fig.

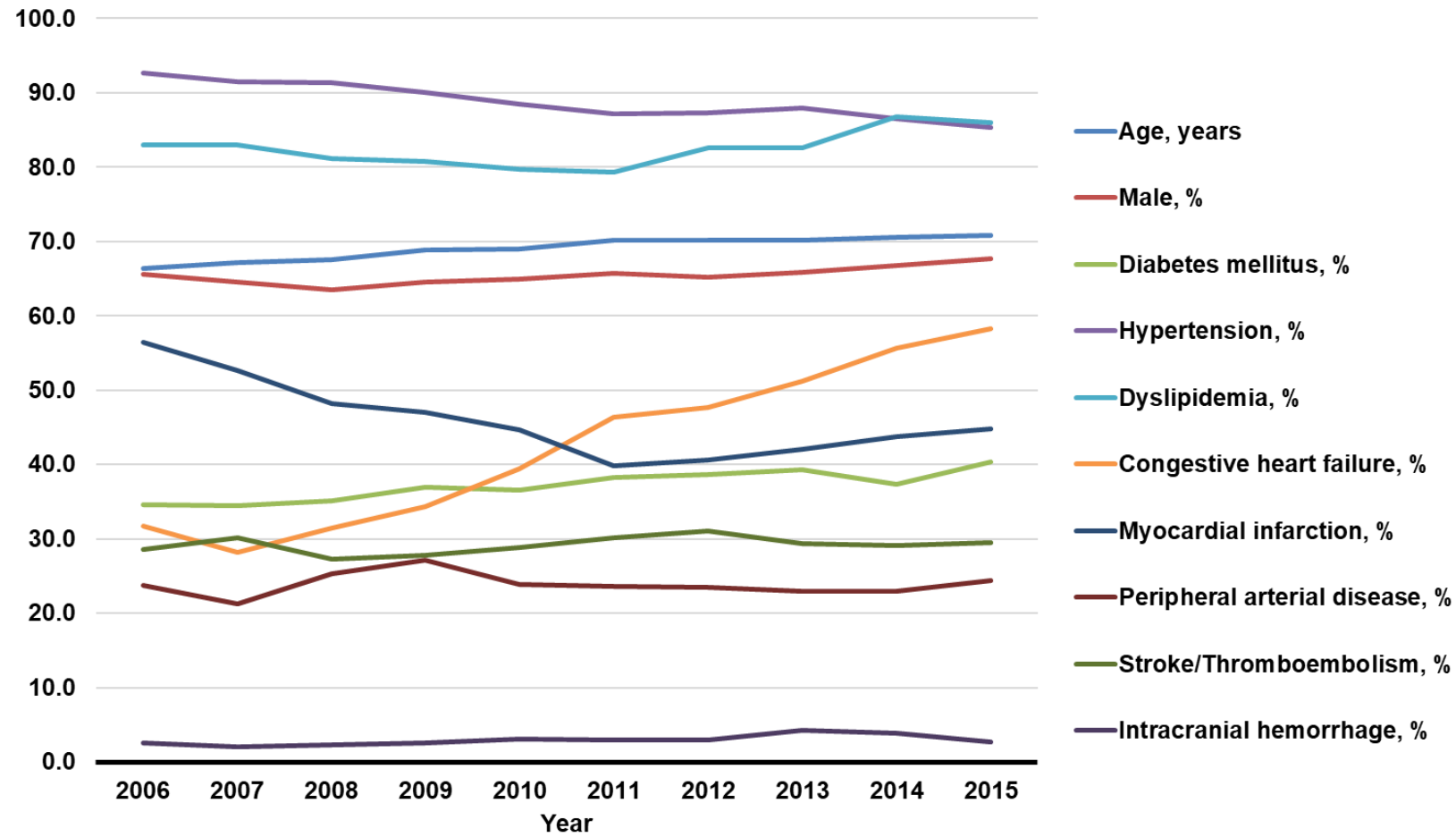

Supplement: S1 Fig — (PDF) [file pone.0209593.s003.pdf]

S2 Fig.

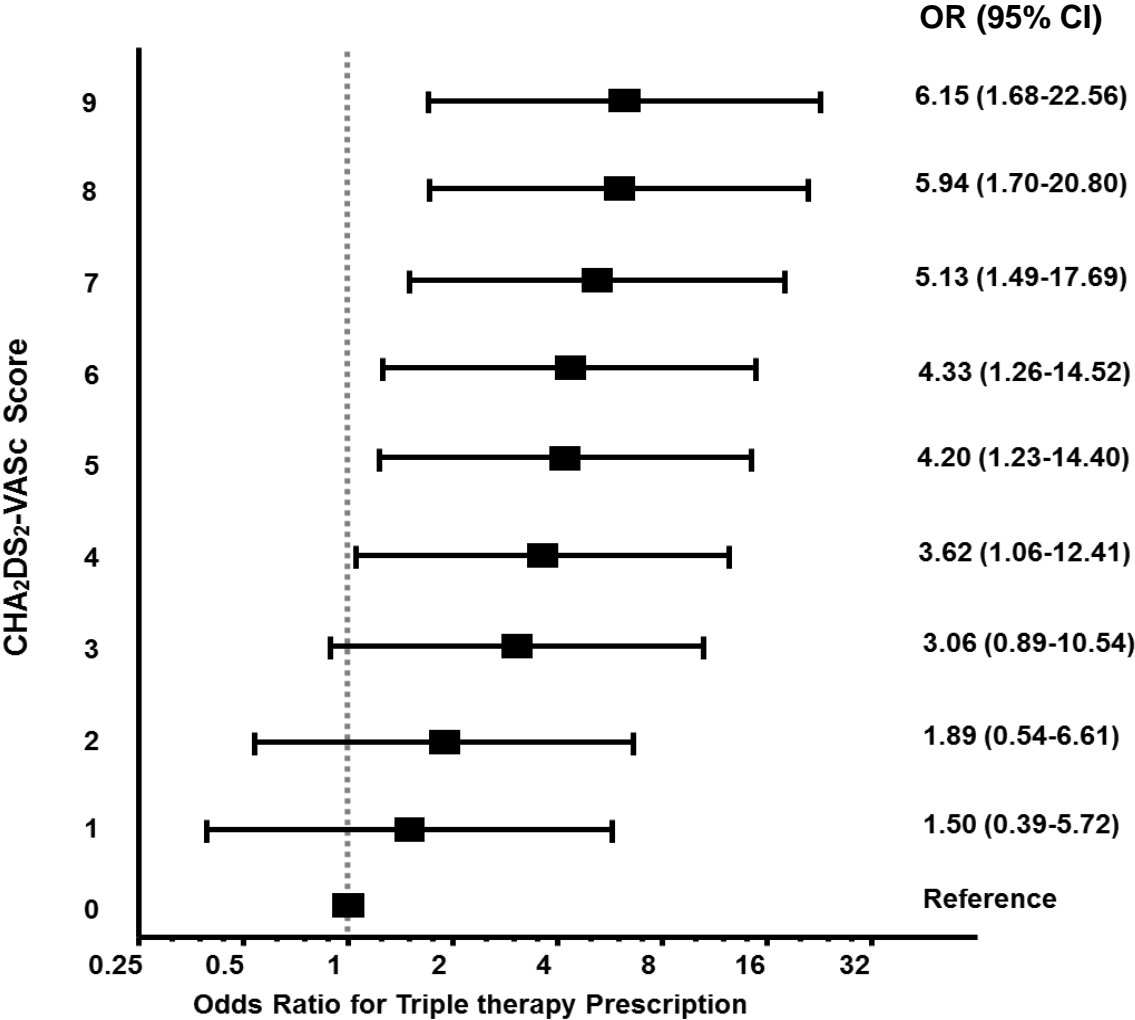

Supplement: S2 Fig — CI, confidence interval; OR, odds ratio (PDF) [file pone.0209593.s004.pdf]
